# Pneumonitis in cancer patients receiving anti-PD-1 and radiotherapies: Three case reports

**DOI:** 10.1097/MD.0000000000005747

**Published:** 2017-01-10

**Authors:** Chieh-Sheng Lu, Jin-Hwang Liu

**Affiliations:** a Department of Internal Medicine, Kaohsiung Armed Forces General Hospital, Kaohsiung; b Division of Hematology/Oncology, Department of Internal Medicine, Tri-Service General Hospital, National Defense Medical Center, Taipei; c Division of Hematology, Department of Medicine, Taipei Veterans General Hospital, Taipei, Taiwan.

**Keywords:** anti-programmed cell death protein 1 (PD-1) treatment, immune-escape, pneumonitis, programmed death-ligand 1 (PD-L1), radiotherapy (RT), upregulation

## Abstract

**Introduction:**

In development of novel therapies for the treatment of patient with cancer, the use of radiotherapy (RT) can produce significant local control and, in recent studies, has also been shown to mediate anti-tumor responses at distant sites by triggering and enhancing the endogenous cellular immune responses. Although RT induces an abscopal effect in some patients due to enhanced immune response to the tumor, immune-escape mechanisms, including up-regulation of programmed death-ligand 1 (PD-L1) on tumor cells, limit this benefit in other patients. Hence, many studies have promoted the synergy of RT and anti-programmed cell death protein 1 (PD-1) treatment for antitumor immunity. However, outcome may be improved when more therapies are combined, but risk of side effects can be increased.

**Case Presentation:**

We herein present 3 advanced cancer patients with pulmonary metastasis and who received RT. Later, they underwent anti-PD-1 treatment and unfortunately suffered from anti-PD-1-related pneumonitis over the nonirradiated areas after 4 cycles of treatment. The upregulation of cellular PD-1 expression in these areas was considered and the immune overreaction by anti-PD-1 treatment may cause these severe pulmonary adverse effects.

**Conclusion:**

Our review of 3 cases warrants careful workup to reduce the risk of side effects by combinative therapy with RT and anti-PD-1 treatment.

## 1 Introduction

Radiotherapy (RT) is widely used in the treatment of primary and metastatic tumors. The inclusion of RT in treatment regimens reduces disease recurrence and improves overall survival in most common cancers.^[1–3]^ In addition to the direct cytoreductive effect, emerging evidence suggests that the generation of antitumor immune responses may play an important role in the effectiveness of RT.^[4,5]^ In the past few years, the new immunotherapies are potent treatment options that have generated a lot of excitement. Antibodies that block the programmed death-ligand 1 (PD-L1) pathway, which cancer cells use to hide from the immune system, include pembrolizumab or nivolumab, anti-programmed cell death protein 1 (PD-1) immunotherapies approved by the Food and Drug Administration (FDA) recently. Notably, the synergistic effects of RT and anti-PD-1 treatment, turning the destroyed tumor cells into a vaccine against the cancer, have become the hot issue in the immunotherapy era. Many trials of PD-1/PD-L1 inhibitors with RT are in development for locally advanced, metastatic cancers and the therapeutic synergy has been considered to improve patient outcomes. However, excessive immune activation may develop and the potential risk of side effects by the combinative therapy is worthy to be investigated. Herein, we presented 3 patients who had received radiotherapy and suffered from immunotherapy-related pneumonitis during anti-PD-1 treatment.

## 2 Case presentation

Approval from our institutional ethics review board was not required for this case report. However, the patients provided written informed consents for the publication of this case report and the accompanying images.

### 2.1 Case 1

**Figure 1:**
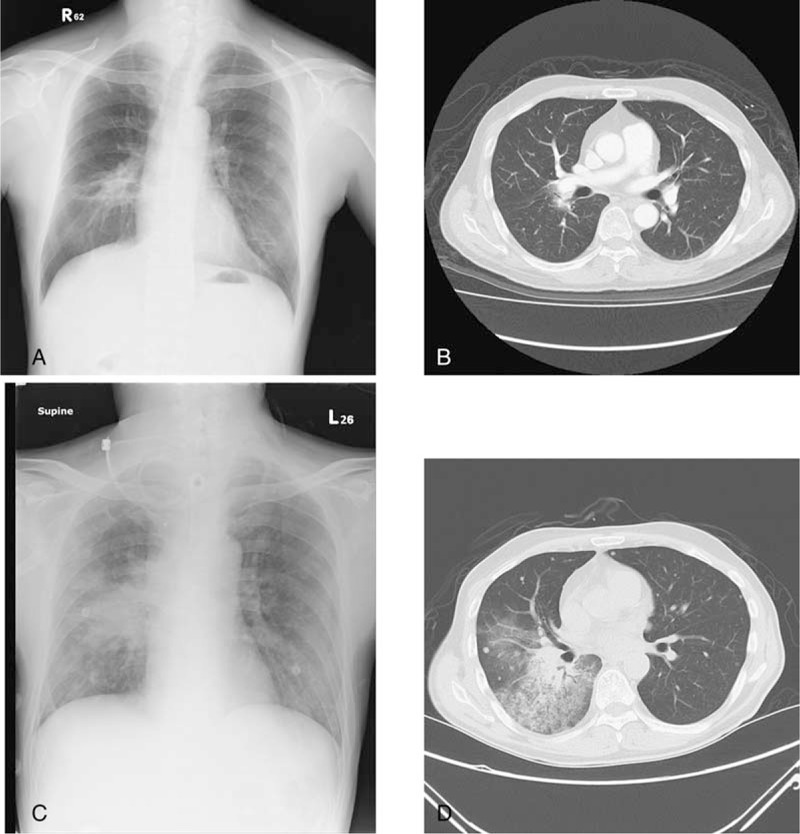
In patient 1, a 54-year-old man with advanced melanoma received a trial of anti-PD-1 treatment with pembrolizumab combined with radiotherapy. Before anti-PD-1 treatment, chest radiograph (CXR) and computed tomography (CT) revealed pulmonary lesions over bilateral hilums (A and B). After 4 cycles of anti-PD-1 treatment, the findings significantly progressed and air-bronchograms at right lower lobe with obstructive pneumonitis was found (C and D). CT = computed tomography, CXR = chest radiograph, PD-1 = programmed cell death protein 1.

A 54-year-old man was diagnosed as having amelanotic melanoma of right middle finger, pT2bN0M0, stage IIA in February 2010, and underwent excisional surgery at that time. Disease recurrence and pulmonary metastasis were developed 1 year later. Local therapies with wedge resection and radiofrequency ablation were done over right lower pulmonary lesions and systemic chemotherapy with dacarbazine (DTIC) plus Proleukin (aldesleukin) were performed. Progressive disease of pulmonary metastasis at bilateral lower lobes was found in February 2014, and he then underwent radiotherapy, total 60 Gy in 20 fractions. During this period, immunotherapy with self-paid ipilimumab was performed since May 24, 2014. Metastatic lymphadenopathy over right anterior neck and newly developed lung lesions (Fig. 1A and B) were still noted 10 months later. Failure of immunotherapy with ipilimumab was considered and he received a trial of anti-PD-1 treatment with pembrolizumab (2 mg/kg, every 3 weeks) from April 23th, 2015. Radiotherapy, total 60 Gy in 15 fractions, was also performed to gross right neck tumors from June 5th, 2015. However, hemoptysis was developed after 4th cycle of anti-PD-1 treatment and chest computed tomography (CT) showed air-bronchograms at right lower lobe with obstructive pneumonitis (Fig. 1C and D). The patient later underwent steroid therapy and anti-PD-1 treatment was on hold.

### 2.2 Case 2

**Figure 2:**
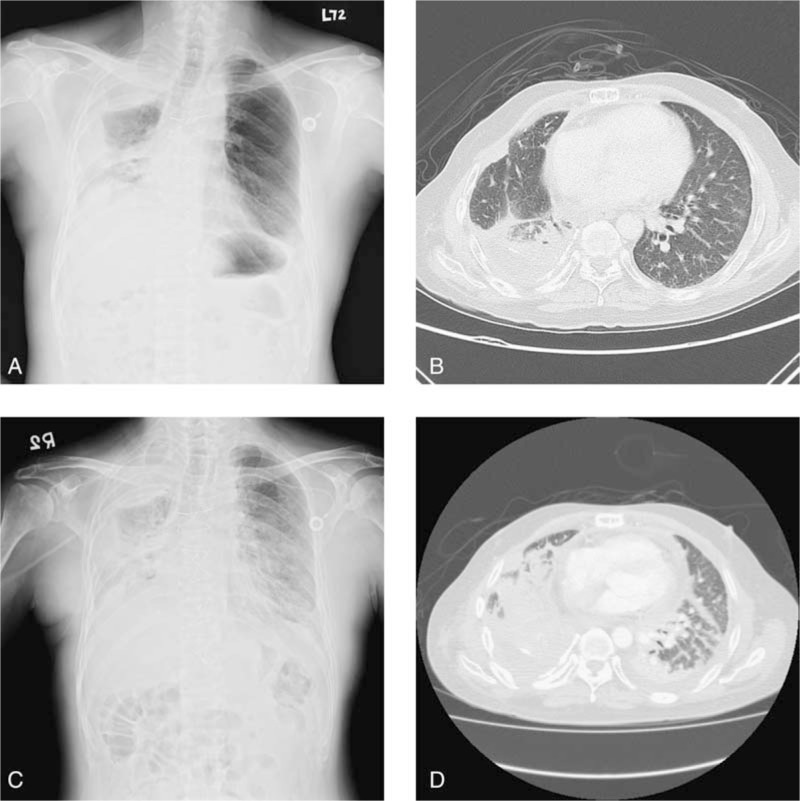
In patient 2, a 57-year-old man with invasive thymoma received photodynamic therapy (PDT) for pulmonary, pleural lesions and followed by the self-paid concurrent treatment with ipilimumab and nivolumab every 3 weeks due to the progressive disease. Before the concurrent immunotherapy, chest radiograph (CXR) and computed tomography (CT) revealed consolidation at right upper, lower lungs, pleural seeding, and lung-to-lung metastasis (A and B). Three months later, the findings significantly progressed and involved all lobes, with decreased lung volumes and pleural effusion (C and D). CT = computed tomography, CXR = chest radiograph, PD-1 = programmed cell death protein 1, PDT = photodynamic therapy.

The patient, a 57-year-old male clinician, was diagnosed as having invasive thymoma, World Health Organization (WHO) type B3, Masaoka stage IV,^[6–8]^ with pleural seeding in September, 2010. Chemotherapy and surgical resection of primary lesions were done in following 2 years. Progressive disease with multiple pulmonary and hepatic nodules was developed 1 year later. He then underwent photodynamic therapy (PDT) for pulmonary, pleural lesions, and radiofrequency ablations for hepatic metastasis. There was less response to the above local treatment and systemic therapy with palliative chemotherapy with DTIC-based regimens was continued from September 2013. Slowly progressive disease (Fig. 2A and B) was still noted in following 1 year and he began to receive the self-paid immunotherapy of ipilizumab combined with chemotherapy from November 2014. Later, the self-paid concurrent treatment with ipilimumab (1 mg/kg) and nivolumab (3 mg/kg, anti-PD-1 regimen) every 3 weeks were prescribed to the patient from February 2015.^[9]^ However, diffuse red plaques over extremities, trunk and face were developed and dyspnea was also noted after 4 cycles of treatment (3 months later). Chest radiograph (CXR) and CT revealed newly developed consolidative lesions in both lungs and fibrosis or pneumonitis was considered (Fig. 2C and D). Anti-PD-1 associated adverse effects were suspected and the treatment was discontinued. Instead of anticancer treatment, the patient received steroid and antibiotic therapies for the above problems. Unfortunately, he suffered from de-saturation and respiratory failure days later because of complications with adult respiratory distress syndrome (ARDS). Finally, the patient refused endotracheal intubation and expired on June 12, 2015.

### 2.3 Case 3

**Figure 3:**
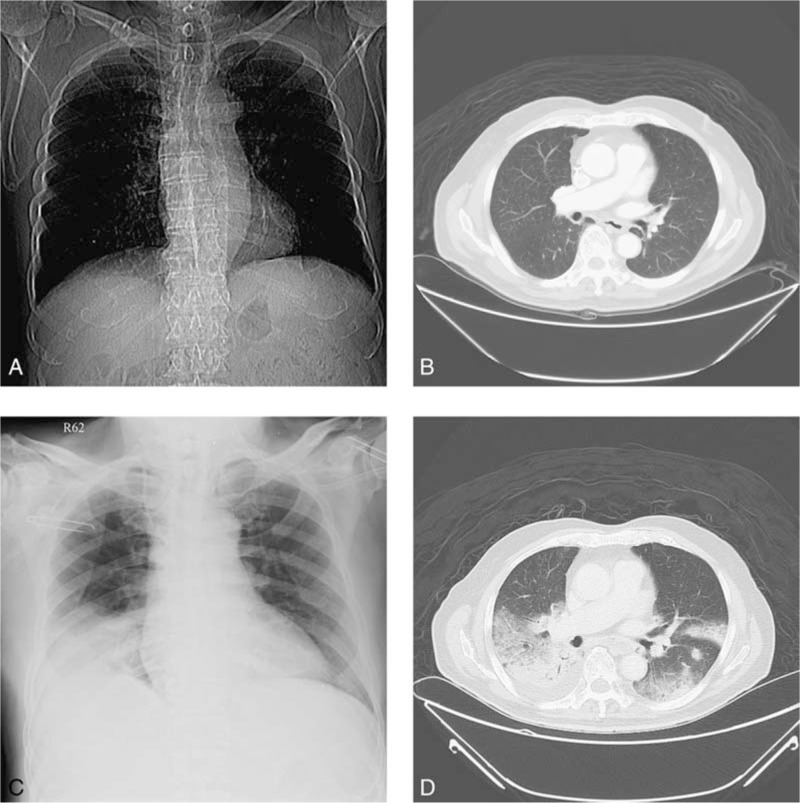
In patient 3, a 79-year-old man with advanced proximal jejunal cancer underwent primary lesions resection and received proton therapy over pulmonary metastatic lesions, followed by the self-paid anti-PD-1 treatment. Before anti-PD-1 treatment, pulmonary metastatic lesions over bilateral hilums were found (A and B). After 4 cycles of treatment, the findings showed multilobular opacities over both lower lungs (C and D). PD-1 = programmed cell death protein 1.

In May 2013, a 79-year-old man with adenocarcinoma of proximal jejunum with splenic flexure colon invasion, carcinomatosis, and lung metastasis visited our hospital. Initially, he underwent left hemicolectomy, small bowel segmental resection for primary lesions, and followed by adjuvant chemotherapy with oxaliplatin-based regimens. Progressive diseases with metastatic foci in right lower lung, lymphadenopathies in para-tracheal, retro-caval, and peritoneal regions were found 6 months later. He then received systemic chemotherapy with irinotecan-based regimens and proton therapy over pulmonary metastatic lesions. Elevated tumor markers were detected (improvement of pulmonary lesions, Fig. 3A and B) and the self-paid salvage immunotherapy with pembrolizumab (2 mg/kg, anti-PD-1 regimen) every 3 weeks was prescribed to him from December 16th, 2014. After 4 cycles of anti-PD-1 treatment, however, the patient suffered from abdominal cramping pain and dyspnea on exertion. CXR showed consolidative patch at right lower lung (Fig. 3C) and abdominal CT revealed intestinal obstruction of distal jejunum, multilobular opacities over both lower lungs (Fig. 3D). He then underwent anterior resection, segmental resection of small intestine, antibiotic treatment, and steroid therapy, and the anti-PD-1 treatment was discontinued due to suspicious anti-PD-1-related pneumonitis.

## 3 Discussion

In cancer patients undergoing RT, the beneficial effects of radiation can extend beyond direct cytotoxicity to tumor cells. Therefore, efforts to improve the efficacy of RT via combination treatment with other treatment regimens could provide clinical benefit. Recent findings indicate that RT induces an abscopal effect in some patients due to the enhanced immune response to the tumor. Evidence suggests that in addition to direct tumoricidal effects, RT can modulate the tumor cell phenotype, increasing antigenicity and sensitivity to immune-mediated killing and induce the release of damage-associated molecular pattern (DAMP) molecules, which enhance the immunogenicity of the tumor by recruiting and making tumor antigens more available to dendritic cells (DC) making the tumor cells more visible to the immune response.^[10–14]^ Despite the immunogenicity of RT-induced tumor cell death, immune-escape still frequently occurs with tumor recurrence. The upregulation of tumor cell PD-L1 expression in response to RT appears to be a mechanism of adaptive resistance by tumor cells limiting the outcome.^[15,16]^ Hence, RT combined with anti-PD-1 treatment has the potential to overcome this resistance and to improve the outcome further. Combination of RT and immune checkpoint inhibitor therapy was effective in mouse models of malignant melanoma or breast cancer xenografts,^[17]^ an orthotopic glioblastoma xenograft model,^[18]^ or colon cancer or breast cancer xenografts.^[19]^ And an improvement of outcome compared to RT or the immune checkpoint inhibitor, alone. Many studies have promoted the synergy of RT and anti-PD-1 treatment for antitumor immunity. However, the risk of side effects with combination therapy may be increased and this issue is more worthy to be investigated. In our 3 patients, they had advanced cancers and received radiotherapy for metastatic pulmonary lesions. To our knowledge, RT can elicit and enhance both the priming and effector phases of antitumor T-cell response. Consequently, if sufficient effector CD8+ cytotoxic lymphocytes are generated, they can migrate to and infiltrate tumors.^[20]^ However, our 3 patients still developed uncontrolled diseases and upregulation of T-cell PD-1 expression was considered. The influence of PD-1 expression compromises the antitumor response. Later, all of these 3 patients received anti-PD-1 treatments and developed newly consolidative lesions over unexpected areas of lungs after 4 cycles of treatment unfortunately. Anti-PD-1-related pneumonitis was considered and excessive immune response was suspected. The phenomenon supported the upregulation of cell PD-1 expression by previous treatments. Thus, greater care should be used upon combination of RT and anti-PD-1 treatment in patients with progressive disease. In the future, precision medicine requires knowing when to give more and what to give.

## 4 Conclusion

In the immunotherapy era, the anti-cancer therapy has been extended the reach of the immune system and many findings are extremely encouraging. However, the adverse effects by the novel combinative therapy should be kept in minds. Herein, we present 3 patients who received RT before anti-PD-1 treatment and suffered from pneumonitis after 4-cycles of anti-PD-1 treatment. Prompt recognition of this rare but severe pulmonary complication in patients is important to ensure successful management of these refractory malignancies with immunotherapy.
